# Personal News Items

**Published:** 1914-10

**Authors:** 


### Personal News Items.

Dr. Longmire of Plainview is now located in Kress.

Dr. F. R. Bassett of Marshall is building a new home.

Dr. J. J. Ingram of Decatur is reported very ill with peritonitis.

Dr. H. F. Blailock and family have recently spent several weeks in Marlin.

Dr. J. D. Clemons of Somerville is able to be up after a brief spell of illness.

Dr. Alva Wright of Houston has returned from attending clinics in the North and East.

Dr. M. L. Kroschel of Hallettsville, has returned from a several months' stay in Europe.

Dr. W. N. Mayfield of Somerville returned on the 5th from a summer tour of Colorado.

Dr. C. G. Strickland of Gustine has formed a partnership with Dr. E. F. Hamm of Clarendon.

Dr. and Mrs. Blassingame of Denison have gone to Chicago, where the doctor will attend clinics.

Dr. H. W. James of Denison has gone to New York to do some special work in the large hospitals there.

Dr. and Mrs. P. M. Payne have recently moved to Texarkana, much to the regret of their Austin friends.

Dr. N. F. Williams of Amarillo has recently been appointed pure food commissioner for the Panhandle.

Dr. E. F. Hughes of Asherton has purchased a home at Uvalde and will soon move there and open an office.

Dr. Winiferd Wilsom of Memphis has been confined to his bed for some time past with a bad case of rheumatism.

Dr. Hulbright of Jayton has recently moved to Ralls, and is reported as doing a lot of business at his new home.

Dr. J. A. Holloway of Round Rock, who has been quite ill, is much improved and is able to be at his office again.

Dr. A. D. Hardin of Dallas has returned from Baltimore, where he has been doing post-graduate work in Johns Hopkins.

Dr. Robert Hasskarl, who has been connected with the Marlin Sanitarium for some time, has gone to Chicago to do post-graduate work.

Dr. P. P. Turner, who has been in Mexico for the past years, has come back to Texas and has opened his office for business in San Marcos.

Dr. and Mrs. John S. Turner are the happy grand-parents of a little grandson, who has been christened John Henry Hudspeth.

Dr. Stephen E. Smith announces that his new sanitarium will be ready for patients by October 15. Crosbyton is to be congratulated on this new addition.

Dr. M. C. Sheppard of Brinker was recently injured when a young horse which he was driving ran away. The injuries while painful were not thought serious.

Dr. A. G. Vick of Belton, who has been ill for the past eight weeks, is much improved. His brother, Dr. Ben Vick, of Valentine, has been with him during his illness.

Dr. J. H. French of Greenville is confined to his home on account of injuries received when his horse became entangled in some wire and threw him out of the buggy.

Dr. Thad Shaw, who for the past two years has been an instructor in the State Medical College at Galveston, has resigned and entered the private practice in San Antonio.

Dr. W. T. White of Dallas has recently been to Baltimore to attend Johns Hopkins. While in the North the doctor visited all the large hospitals of Chicago, New York and Philadelphia.

Dr. M. H. E. Whitesides of Timpson has returned from Hot Springs, Ark., where he went for the benefit of his health. The doctor is much improved and will take up his work again soon.

The medical firm of Samuell, Block, Thomasson & Hill of Dallas are planning a three-story office building, which is to cost $30,000. A modern laboratory will be part of the equipment of the building.

Dr. J. E. Warner, while treating a case of charbon, became infected. He was carried to a sanitarium and the place infected was cauterized. The attending physicians think that he will fully recover.

Dr. W. T. Thackery of Fowlerton has returned from Chicago, where he had a cancer of the tongue removed. The operation was a successful one and the doctor is well again, much to the delight of his many friends.

Dr. E. H. Cary, dean of the medical department of the Baylor University, has just returned from attending the Congress of Surgeons in London. The doctor says that he purchased passage on three different steamers before he struck the one that sailed. He says that the thoughtful Englishmen feel that it is going to take a long time to whip Germany, but that they will do it eventually. He adds, however, that the Germans are the most scientific people in the world and that they are fighting along scientific lines.

### Change of Address.

Dr. J. A. Allen from Fulbright to Detroit.
Dr. T. R. Burnett from Carrelton to Mission, Texas.
Dr. N. E. Dick from Nashville, Tenn., to Milsap, Texas.
Dr. J. W. Mickle from Sulphur, Okla., to Clarendon, Texas.
Dr. H. L. D. Kirkham from Brownsville to Houston.
Dr. J. E. Rhymes from Houston to Blum.
Dr. W. A. Reed from Angleton to Burton.
Dr. H. B. Pender from Jacksonville to Greenville.
Dr. P. M. Payne from Austin to Texarkana.
Dr. L. A. Patterson from Sabinal to Medina City.
Dr. C. J. Logan from Silver City, N. M., to El Paso, Texas.
Dr. T. A. Morrison from Santa Anna to Grosvenor, Texas.

### Here and There Among the Medical Societies.

On the morning of October 8, at 10 o'clock, the meeting of the thirty-sixth semi-annual South Texas District Medical Association was called to order in the lecture hall of the St. Mary's Infirmary, Galveston, Texas. The component county societies are those of the Eighth, Ninth and Tenth Districts. The program is too long to publish in full, but it is most interesting, and some of the most prominent physicians in the State read and discussed papers. There was a section on medicine and diseases of children, one on obstetrics and gynecology, and one on surgery.

Dr. C. C. Bass, formerly of Galveston but now of New Orleans, delivered an address on "Pyorrhea Alveolaris," setting forth the great danger and the prevalence of this disease, which causes 90 per cent of the human family to lose their teeth.

Dr. Bass said that a solution of ementine hydrochloride was effective in healing this disease, which has heretofore been thought incurable.

Many other excellent papers were read during the meeting.

One of the main features on the program was a public health meeting on the evening of the 8th, to which the public was invited.

Dr. Paschal of San Antonio, Professor Horne of the Houston High School, and Dr. James Terrell of Temple were the speakers of the evening.

After adjournment of the scientific session of the first day there was an automobile ride over the city, and after the ride a "sea food supper."

The Bowie and Miller County Medical Societies will hold their regular meeting in Texarkana on the 23rd of October. Dr. Rodney Dale will read a paper on "Ductless Glands," and Dr. B. E. Dixon will read a paper on "Extra-Peritoneal Caesarian Section."

The next meeting of the Nacogdoches County Medical Society will be held in Nacogdoches October 14. A very interesting program is promised.

The Delta County Medical meeting was held in Cooper and a very interesting program was carried out. Dr. Gregory read a paper on the "Cause and Prevention of Insanity," which was much appreciated, and the doctor was given a vote of thanks.

The regular monthly meeting of the Hill County Medical Association was held in Hillsboro. There were thirty members present. Dr. O. F. Goeber of Temple read a very interesting paper. Dr. Wood of Hubbard gave an interesting report of his trip abroad. Dr. Ivey of Whitney made a report of a very unique clinic, and Holland presented to the association a new parasite for microscopical examination.

The quarterly meeting of the Bell County Medical Association was held in Belton, September 2. Between forty and fifty members were present. A thorough discussion was made of the cause and prevention and treatment of dysentery and kindred diseases. After the business was finished the doctors were served delicious fruit and cream.

The Travis County Medical meeting met in regular session September 27. Interesting and instructive talks were made by Dr. John Preston, Dr. E. E. Suehs, and Dr. R. V. Murray. Enjoyable refreshments were served.

The bulletin of the Hood-Somervell Medical Society is the most unique report that comes to our dask. This month it contains a pen picture of the secretary hunting for the members of the society with a lighted lantern. If all the members of the society were as faithful and unselfishly devoted to the upbuilding of the society as is the secretary they would have standing room only.

A largely attended and enthusiastic meeting of the Navarro County Medical Society was held in Corsicana on September 7. Many of the doctors came from other towns and all felt that the meeting was most profitable. The discussions were animated and helpful.

The Fifth and Sixth District Medical Society, a merger of the Bexar and Nueces County Medical Societies, held its first meeting in Corpus Christi, Texas, Wednesday and Thursday, September 8 and 9. The following physicians were in attendance and took an active part in the discussions of the various papers read, and in the festivities provided for their entertainment, viz:

Corpus Christi—Drs. W. N. Wardlow, F. N. Painter, R. H. L. Bibb, P. D. Spohn, Herbert Caldwell, L. Kaffie, C. P. Yeager, T. J. Turpin, D. T. Stanley, W. L. Peters, A. J. Caldwell, Jos. D. Cohn, C. M. Payne, G. M. Abney, E. O. Arnold, J. J. Robertson, S. T. Dodge, Harry Heaney, A. D. Davisson, Geo. Gregory, W. E. Carruth.

San Antonio—Drs. J. S. Langford, J. A. McIntosh, J. H. Bigger, D. M. Stone, S. P. Cunningham, D. S. Edwards, Chas. S. Venable, W. B. Russ, L. L. Shropshire, J. H. Burleson, G. H. Baker, F. E. Young.

Houston—Drs. W. Burton Thorning, Charles M. Aves, Wallace Ralston.

Dr. L. J. Manhoff, Dr. Walter Noble, Aransas Pass; Dr. W. T. Dunning, Gonzales; Dr. H. Rushing, Runge; Dr. E. H. Sauvignet, Laredo; Dr. N. A. Poth, Seguin; Dr. R. C. Youngblood, Falls City; Dr. A. T. Hightower, Odem; Dr. Wm. Lee Secor, Kerrville; Dr. J. W. James, Calallen; Dr. M. J. Perkins, Alice; Dr. Stanley Whitacre, Sinton; Dr. L. M. Davis, Robstown; Dr. W. H. Smith, Hondo; Dr. Chas. Wendelken, Gregory; Dr. Frank E. Osborn, McAllen; Dr. L. E. Devendorf, Taft.

The following list of papers, which were generally discussed, were read: "Use of Forceps," by Dr. T. I. Turpin, Corpus Christi; "Repair of Perineal Tears," by Dr. W. B. Thorning, Houston; "Some of the End Results Following Lacerations from Childbirth," by Dr. F. E. Young, San Antonio; "Procidentia Uteri," by Dr. P. D. Spohn, Corpus Christi; "Painless Childbirth," by Dr. W. L. Peters, Corpus Christi; "Rabies," by Dr. R. H. L. Bibb, Corpus Christi; "Report of a Case of Syphiloma of the Liver with Ascites and Lientery," by Dr. Joe D. Cohn, Corpus Christi; "Risks to Life Insurance Companies from Heart Disease," by Dr. I. B. Langford, San Antonio; "Involution Melancholia," by Dr. I. A. McIntosh, San Antonio; "Pathology and Treatment of Sprained Ankle," by Dr. G. H. Baker, San Antonio; "Why the Country Practitioner Should not do Major Surgery," by Dr. R. C. Youngblood, Falls City; "Intestinal Stasis from a Surgical Standpoint," by Dr. W. B. Russ, San Antonio; "Diseases of the Gall Bladder and Their Treatment," by Dr. C. P. Yeager, Corpus Christi; "Traumatic Hernia," by Dr. S. P. Cunningham, San Antonio; "Pelvic Reflexes and Their Relation to Ocular Disturbances," by Dr. I. H. Burleson, San Antonio; "Relation of Infectious Diseases to the Ear, Nose, and Throat," by Dr. D. S. Edwards, San Antonio; "Some Pathological Conditions in the Nose and Pharynx and Their Effects on the Human Economy."

This meeting was one of the liveliest and most interesting assemblage of medical men of the many that it was ever my good fortune to enjoy, and the program was carried through to the satisfaction of everyone who attended.

The newly elected officers, all of them by acclamation, are: Dr. J. A. McIntosh of San Antonio, President; Dr. E. H. Sauvignet of Laredo, Vice President, and Dr. L. J. Manhoff of Aransas Pass, Secretary (re-elected). They are too well known to require an introduction to the public. Suffice it to write that their names, as officers of the society, make sure that the next meeting, to be held at San Antonio in December, will be as successful and as interesting as the one just closed here.

R. H. L. BIBB.

